# Supplementary material for: No Evidence of Reactive Avoidance of Baboons ( Papio ursinus and Papio anubis ) to the Presence of Predators
Source: Ecol Evol. 2026 May 5;16(5):e73609. doi: 10.1002/ece3.73609 (PMC13143513; doi:10.1002/ece3.73609)

**Supplementary Figure 1**. Distribution of significance values (*p*) from the randomization test assessing the effect of lions (*Panthera leo*) on Burchell’s zebra (*Equus quagga*) detections in Associated Private Nature Reserves (APN), South Africa (data collected between 2017 and 2019) within the 0 to 24 hours following lion detection. The test was conducted on randomly down-sampled Burchell’s zebra detections data sets (n = 100). The red dashed line indicates the significance value threshold of alpha = 0.05, marking the significance level for detecting deviations from the assumption of non-avoidance.


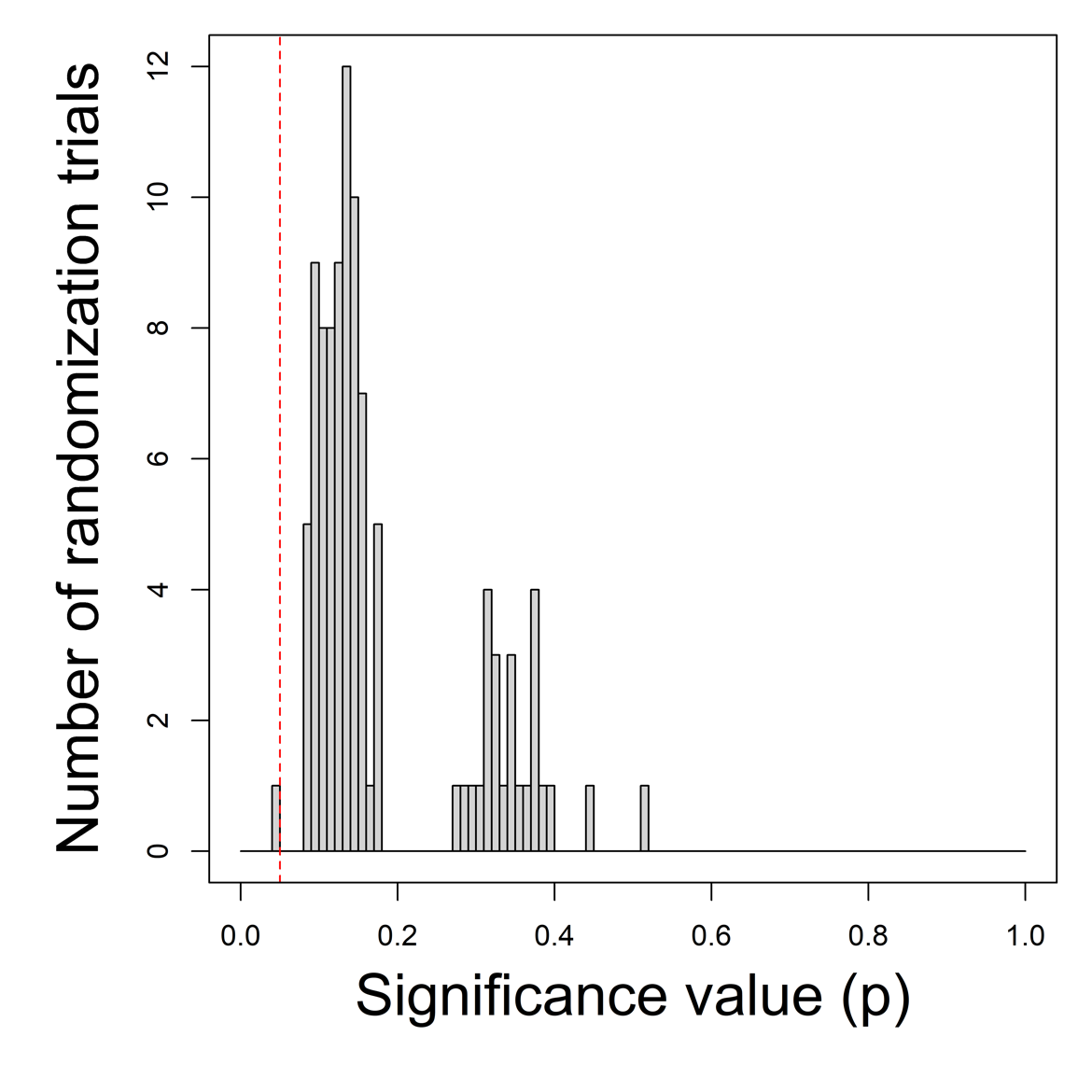

Supplement: Supplementary file 1 — Figure S1: Distribution of significance values (p) from the randomization test assessing the effect of lions ( Panthera leo ) on Burchell's zebra ( Equus quagga ) detections in Associated Private Nature Reserves (APN), South Africa (data collected between 2017 and 2019) within the 0–24 h following lion detection. The test was conducted on randomly down‐sampled Burchell's zebra detections data sets (n = 100). The red dashed line indicates the significance value threshold of alpha = 0.05, marking the significance level for detecting deviations from the assumption of non‐avoidance. [file ECE3-16-e73609-s001.docx]
